# Diabetic ketoacidosis and hyperglycemic hyperosmolar state are associated with higher in-hospital mortality and morbidity in diabetes patients hospitalized with ST-elevation myocardial infarction, but not within 30 days of readmission

**DOI:** 10.1371/journal.pone.0318774

**Published:** 2025-02-06

**Authors:** Turki Almutairi, Soha Dargham, Amin Jayyousi, Jassim Al Suwaidi, Charbel Abi Khalil

**Affiliations:** 1 Research Department, Weill Cornell Medicine-Qatar, Doha, Qatar; 2 Biostatistics Core, Weill Cornell Medicine-Qatar, Doha, Qatar; 3 Department of Medical Education, Weill Cornell Medicine-Qatar, Doha, Qatar; 4 Department of Endocrinology, Hamad Medical Corporation, Doha, Qatar; 5 Heart Hospital, Hamad Medical Corporation, Doha, Qatar; 6 Joan and Sanford I, Weill Department of Medicine, Weill Cornell Medicine, New York, United States of America; Pelita Harapan University Faculty of Medicine: Universitas Pelita Harapan Fakultas Kedokteran, INDONESIA

## Abstract

**Background:**

While the cardiovascular risk of hyperglycemia has been thoroughly elucidated in patients with type 2 diabetes (T2DM) hospitalized for myocardial infarction, the evidence surrounding acute severe hyperglycemia is less well-established. Our study aimed to explore the impact of diabetic ketoacidosis (DKA) and hyperglycemic hyperosmolar state (HHS), both severe hyperglycemic conditions, on cardiovascular outcomes in patients with T2D admitted for ST-elevation myocardial infarction (STEMI).

**Methods:**

We used the National Readmission Database (2016–2019) to extract patients with T2DM and STEMI at baseline. Subsequently, we selected cases of DKA and HHS. The primary endpoint was in-hospital mortality. Secondary endpoints included in-hospital acute renal failure, cardiogenic shock, and 30-day readmission and mortality.

**Results:**

The presence of DKA increased the adjusted odds of mortality and cardiogenic shock by almost 2-fold (adjusted Odds Ratios aOR = 2.30 [1.70–3.12], 2.055 [1.602–2.637], respectively) and renal failure by nearly 5-fold (aOR = 5.175 [4.090–6.546]). HHS was also associated with higher odds of mortality, acute renal failure, and cardiogenic shock. In 30 days, DKA and HHS increased the risk of readmission (aOR = 1.815 [1.449–2.75], 1.751 [1.376–2.228], respectively). There were no differences in the rates of cardiovascular disease, mortality, or other cardiovascular events between DKA and HHS patients. Within 30 days of readmission, DKA and HHS were associated with higher odds of readmission but not mortality. Cardiovascular disease was the most common etiology of readmission in all patients. The incidence of non-STEMI was the highest in DKA patients, and the incidence of STEMI was the highest in the HHS group.

**Conclusion:**

The presence of diabetic ketoacidosis or hyperglycemic hyperosmolar state is associated with higher odds of mortality, renal failure, cardiogenic shock, and 30-day readmission in STEMI patients with type 2 diabetes, highlighting the need for enhanced clinical management and monitoring of patients experiencing acute hyperglycemia.

## I. Introduction

Type 2 diabetes mellitus (T2DM) is a complex pathophysiological condition involving metabolic dysregulation of blood glucose homeostasis [1]. It is characterized by insulin resistance progressing toward pancreatic beta-cell dysfunction [2]. The burden of this disease is reflected in its financial implications and sizeable contribution to the global health crisis. The International Diabetes Federation estimated that the total number of patients with diabetes will increase to 642 million by 2040, with an equally alarming total global health expenditure cost of US$802 billion [3]. In addition, 5 million deaths in adults aged 20–79 in 2015 were attributed to diabetes, accounting for 12.8% of global all-cause mortality [3].

Cardiovascular disease (CVD) is the leading cause of mortality in patients with diabetes [4]. Patients with T2DM are at risk of significant cardiovascular events as well as an increased mortality risk [2]. One of the most important manifestations of CVD in patients with diabetes is coronary artery disease (CAD). Moreover, T2DM is considered a coronary artery disease equivalent, comparable to a prior CAD history [5]. Studies have shown a higher incidence of coronary atherosclerosis and stenosis in patients with diabetes compared to those without the condition [3]. In particular, diabetes accounts for a substantial mortality burden among patients admitted with myocardial infarction, particularly ST-elevation myocardial infarction (STEMI) [6].

Diabetic ketoacidosis (DKA) and hyperglycemic hyperosmolar state (HHS) are acute, life-threatening hyperglycemic conditions [7]. Both DKA and HHS can cause significant electrolyte disturbances, such as hypokalemia or hyperkalemia, which predispose patients to arrhythmias, including atrial fibrillation and ventricular tachycardia [8]. Further, hyperglycemia, dehydration, and associated metabolic stress in DKA and HHS can lead to decreased coronary perfusion, resulting in myocardial ischemia [9]. Furthermore, profound electrolyte imbalances and arrhythmias in the context of DKA and HHS can lead to sudden cardiac death [8].

We have previously demonstrated that hypoglycemia in STEMI patients is associated with a higher risk of mortality and arrhythmias [10]. The relationship between chronic hyperglycemia and adverse clinical outcomes in patients with acute STEMI is well-established, even in those without diabetes [11]. However, there is a lack of studies assessing the impact of acute hyperglycemia. This study aimed to evaluate the impact of diabetic ketoacidosis (DKA) and hyperglycemic hyperosmolar state (HHS) on STEMI with type 2 diabetes.

## II. Methods

### a. Study design and database sampling

We extracted data from the National Readmission Database (NRD), which was developed as part of the Healthcare Cost and Utilization Project (HCUP) by the Agency for Healthcare Research and Quality. The NRD is the largest inpatient database that includes information regarding hospital readmissions regardless of payer status. It provides important data from 17 million discharges, allowing for analyses of readmission at the US national level, and it is updated yearly. The NRD includes data using ICD-10 codes (as of 2016) for different diagnoses and procedures and uses patient linkage numbers to track patient hospitalizations across various facilities. It provides de-identified patient information regarding discharged patients with or without repeat hospital visits. In our study, we collected data between 2016 and 2019. The ethics and research committees at Weill Cornell Medicine-Qatar approved the research project with IRB approval (record number 21–00021). Data was accessed on 29/08/2023. It was de-identified, and the authors did not have access to information that could identify individual participants during or after data collection. Patient consent was not needed.

### b. Outcomes and measures

We included STEMI patients (primary diagnosis) with type 2 diabetes (secondary diagnosis) in our analysis. We stratified patients according to the development of DKA and HHS during hospitalization (tertiary diagnosis). We compared patients with DKA and HHS to those who did not develop either of those conditions. We compared patients with DKA to those with HHS. Finally, we assessed outcomes at 30-day readmission in the same patients. The primary endpoint was in-hospital mortality. Secondary endpoints included acute renal failure, cardiogenic shock, and 30-day readmission and mortality. We further assessed the incidence of mortality and readmission, the underlying etiology, and the predictors of both outcomes.

### c. Statistical analyses

The baseline characteristics of patients are presented as mean (SD), median (IQR), or number (%). The comparison was done using the student t-test, Chi-square tests, and Fisher’s exact tests as deemed appropriate. A binary logistic regression assessed cardiovascular events presented as odds ratios (OR) with their 95% confidence intervals. Further, the risk was adjusted on age, sex, and statistically significant parameters between groups at the univariate level. A multivariate logistic regression model was performed to determine predictors of mortality and readmission. Significance was defined at the 5% level. Statistical analyses were conducted using IBM-SPSS.

## III. Results

### a. Study population

**Fig 1:**
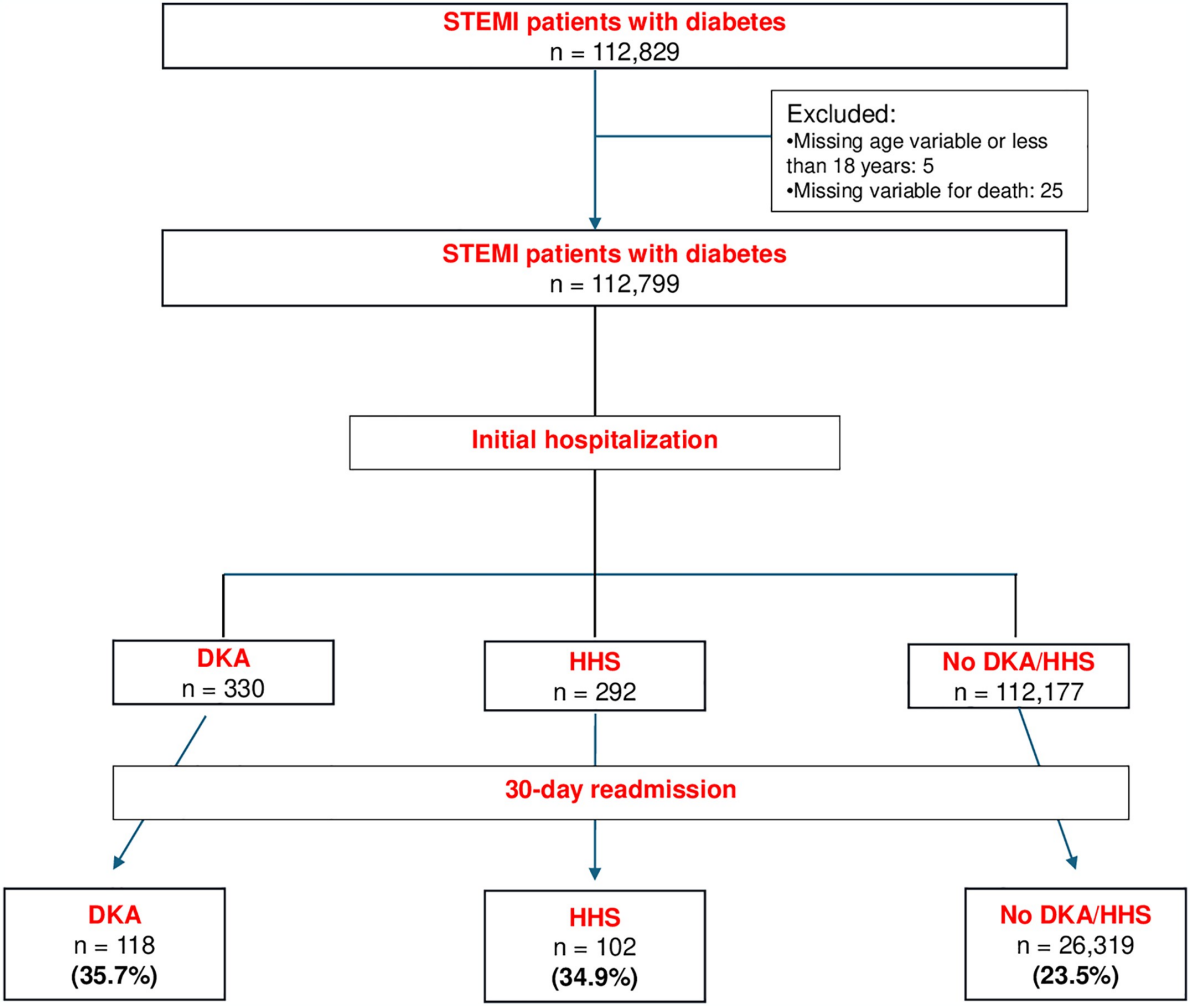
Flow chart of the study.

A total of 112,829 patients hospitalized for STEMI with a concurrent diagnosis of type 2 diabetes were hospitalized between 2016 and 2019, of whom 112,799 were included in our analysis after excluding patients with missing data (Fig 1). Of those patients, 330 had a diagnosis of DKA, and 292 had a diagnosis of HHS. At 30 days following the initial hospitalization, 118 (35.7%) of the initial DKA group, 102 (34.9%) of the initial HHS group, and 26,523 (23.6%) of STEMI patients with diabetes were readmitted.

### b. In-hospital outcomes

#### Diabetic ketoacidosis

DKA patients were younger than non-DKA patients (53.29 [±14.57] years vs 64.60 [±12.46] years, respectively, p<0.001) and were more likely to be female (46.7% compared to 34.2%, p<0.001). Conversely, DKA patients were less likely to have obesity, dyslipidemia, and chronic kidney disease (CKD) (p<0.001 for all) (Table 1). However, the presence of DKA increased the odds of mortality and cardiogenic shock by almost 2-fold (adjusted OR aOR = 2.30 [1.698–3.117], 2.055 [1.602–2.637], respectively) and acute renal failure by nearly 5-fold (aOR = 5.175 [4.090–6.546]) (Table 2).

**Table 1:** Comparison of demographics between index patients with STEMI, with DKA, HHS, and without DKA/HHS.

		DKA	HHS	Non DKA/HHS	P-value
**Age**	Mean (SD)	53.29 (14.569)	64.18 (12.697)	64.60 (12.456)	*, ***
	<55	177 (53.6%)	65 (22.3%)	24380 (21.7%)	
	55–64	71 (21.5%)	79 (27.1%)	32131 (28.6%)	
	65–74	60 (18.2%)	88 (30.1%)	30702 (27.3%)	
	75–84	18 (5.5%)	38 (13.0%)	17971 (16.0%)	
	>84	4 (1.2%)	22 (7.5%)	7285 (6.5%)	
**Gender**	Male	176 (53.3%)	158 (54.1%)	74051 (65.8%)	*,**
	Female	154 (46.7%)	134 (45.9%)	38418 (34.2%)	
**Income**	Low	90 (27.9%)	89 (30.9%)	32693 (29.5%)	***
	Low-Mid	75 (23.2%)	92 (31.9%)	31159 (28.2%)	
	High-Mid	91 (28.2%)	66 (22.9%)	27177 (24.6%)	
	High	67 (20.7%)	41 (14.2%)	19608 (17.7%)	
**Comorbidities**	Obesity	39 (11.8%)	69 (23.6%)	29766 (26.5%)	*,***
	Smoking	62 (18.8%)	35 (12.0%)	23064 (20.5%)	**,***
	Dyslipidemia	168 (50.9%)	182 (62.3%)	79393 (70.6%)	*,**,***
	Hypertension	221 (67.0%)	243 (83.2%)	96475 (85.8%)	*, ***
	PVD	27 (8.2%)	31 (10.6%)	8790 (7.8%)	
	VHD	30 (9.1%)	487 (10.1%)	11276 (10.0%)	**
	CKD	106 (32.1%)	94 (32.2%)	23749 (21.1%)	*,**
	CAD	232 (70.3%)	213 (72.9%)	83001 (73.8%)	

* P<0.05 DKA vs non DKA/HHS.

** P<0.05 HHS vs non DKA/HHS.

*** P<0.05 DKA vs HHS.

CKD = chronic kidney disease, PVD = peripheral vascular disease, VHD = valvular heart disease.

**Table 2:** Comparison of both in-hospital and 30-day outcomes between STEMI patients with DKA, HHS, and without DKA/HHS.

	In-hospital outcome			30-day outcome	
	In-hospital mortality (aOR, 95% CI)	Cardiogenic shock (aOR, 95% CI)	Acute renal failure (aOR, 95% CI)	Readmission (aOR, 95% CI)	Mortality (aOR, 95% CI)
**DKA vs** **non-DKA/HHS**	2.30 (1.698–3.117)	2.055 (1.602–2.637)	4.495 (3.616–5.589)	1.815 (1.449–2.75)	0.988 (0.361–2.704)
**HHS vs** **non DKA/HHS**	1.602 (1.147–2.237)	2.684 (2.091–3.445)	4.567 (3.623–5.758)	1.751 (1.376–2.228)	1.617 (0.743–3.516)
**DKA vs HHS**	0.675 (0.410–1.109)	1.305 (0.918–1.855)	1.017 (0.740–1.396)	0.964 (0.693–1.340)	3.529 (0.560–22.244)

#### Hyperglycemic hyperosmolar state

There were no mean age or distribution differences between patients with HHS and those without (Table 1). The HHS group had a more significant proportion of females, a higher prevalence of chronic kidney disease (CKD), and valvular heart disease (VHD), but a lower prevalence of smoking and dyslipidemia (p<0.001 for all). HHS was associated with higher odds of mortality (aOR = 1.602 [1.147–2.237]), cardiogenic shock (aOR = 2.466 [1.910–3.186]), and acute renal failure aOR = 4.567 [3.623–5.758]). (Table 2).

#### Comparison of diabetic ketoacidosis to hyperglycemic hyperosmolar state

DKA patients were, on average, younger by approximately ten years compared to their counterparts with HHS (53.29 [14.57] years compared to 64.18 [12.697] years, respectively, p<0.001) (Table 1). There were no differences in the gender distribution, but both groups had a higher proportion of male patients. DKA patients were more likely to have a higher income, while the prevalence of obesity, dyslipidemia, and hypertension was lower compared to the HHS group (p<0.05 for all). There was no difference in the odds of mortality and other cardiovascular endpoints (Table 2).

### c. Readmission

#### Diabetic ketoacidosis

Out of the initial DKA group, 118 (35.7%) were readmitted, compared to 26,523 (23.6%) STEMI patients with diabetes but without DKA or HHS. Patients who were readmitted were, on average, 13 years younger (53.4 [14] vs 66 [12], p<0.001), had a higher proportion of females (50.8% vs 40.4%, p = 0.002), and were less likely to be less obese (Table 3). DKA was associated with a higher risk of 30-day readmission (aOR = 1.815[1.449–2.75]). However, 30-day mortality was not significantly different between the groups (Table 2).

**Table 3:** Comparison of characteristics between readmission STEMI patients with DKA, HHS, and without DKA/HHS.

		DKA	HHS	Non DKA/HHS	P-value
**Age**	Mean (SD)	53.44 (14.052)	63.43 (12.348)	66.32 (12.508)	*
	<55	66 (55.9%)	30 (29.4%)	5430 (20.5%)	*,**,***
	55–64	26 (22.0%)	19 (18.6%)	6273 (23.7%)	
	65–74	20 (16.9%)	36 (35.3%)	7480 (28.2%)	
	75–84	5 (4.2%)	11 (10.8%)	5240 (19.8%)	
	>84	1 (0.8%)	6 (5.9%)	2100 (7.9%)	
**Gender**	Male	58 (49.2%)	56 (54.9%)	15797 (59.6%)	*
	Female	60 (50.8%)	46 (45.1%)	10726 (40.4%)	
**Income**	Low	38 (32.2%)	29 (29.0%)	8374 (32.0%)	
	Low-Mid	32 (27.1%)	37 (37.0%)	7425 (28.4%)	
	High-Mid	25 (21.2%)	21 (21.0%)	6202 (23.7%)	
	High	23 (19.5%)	13 (13.0%)	4185 (16.0%)	
**Comorbidities**	Obesity	14 (11.9%)	18 (17.6%)	5923 (22.3%)	*
	Smoking	23 (19.5%)	29 (28.4%)	6766 (25.5%)	
	Dyslipidemia	62 (52.5%)	75 (73.5%)	17917 (67.6%)	*
	Hypertension	100 (84.7%)	93 (91.2%)	23754 (89.6%)	
	PVD	15 (12.7%)	11 (10.8%)	2749 (10.4%)	
	VHD	7 (5.9%)	8 (7.8%)	3079 (11.6%)	
	CKD	42 (35.6%)	34 (33.3%)	9367 (35.3%)	
	CAD	82 (69.5%)	67 (65.7%)	19768 (74.5%)	

* P<0.05 DKA vs non DKA/HHS.

** P<0.05 HHS vs non DKA/HHS.

*** P<0.05 DKA vs HHS.

#### Hyperglycemic hyperosmolar state

A total of 102 (34.9%) out of the initial HHS group were readmitted, compared to 26,523 (23.6%) STEMI patients with diabetes but without DKA or HHS. In comparing groups, no differences were observed in baseline characteristics or mean age. However, there was a statistically significant difference in the age distribution as the proportion of patients over 65 was higher in the HHS group (Table 3). Within 30 days of initial hospitalization, HHS was associated with higher odds of readmission (aOR = 1.751[1.376–2.228]) but not mortality (Table 2).

#### Comparison between DKA and HHS

Readmitted patients with DKA had a lower rate of dyslipidemia (52.5% vs. 73.5%, p = 0.001) than HHS patients (Table 3). The readmission rate was the same for both groups (48.3% vs. 50.0%, DKA vs. HHS, respectively, p = 0.892). Within the 30-day readmission cohort, the odds of mortality were similar between the two groups (Table 2).

### d. Etiology of readmission

**Fig 2:**
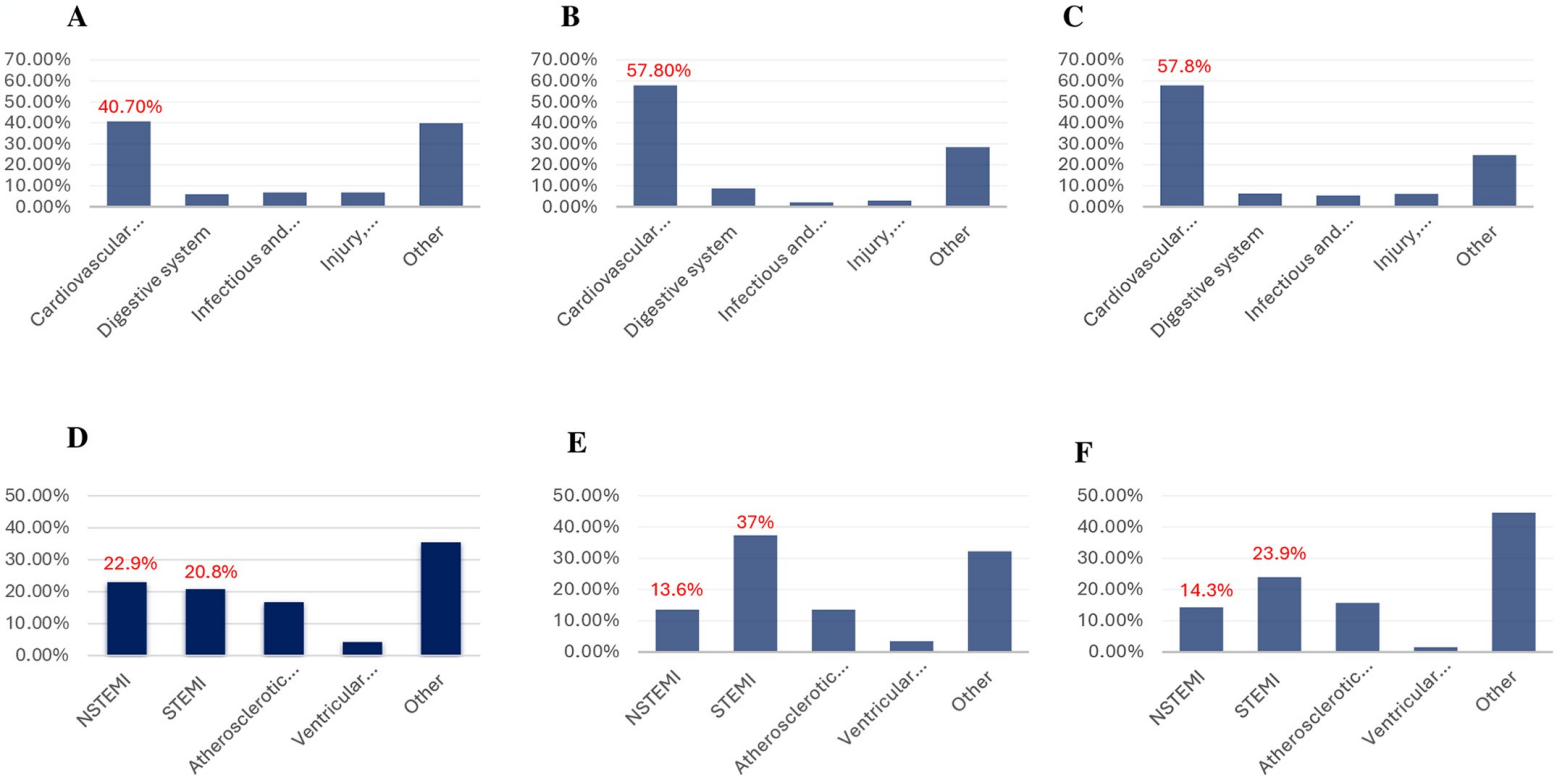
Most common etiologies of readmission in patients with (**A**) DKA, (**B)** HHS, and (**C**) Non-DKA/HHS. The most common cardiovascular diagnoses in readmitted patients with (**D**) DKA, (**E**) HHS, and (**F**) Non-DKA/HHS.

We explored the top five etiologies of readmission in all groups. Cardiovascular pathology was the most common etiology of readmission across all groups (Fig 2A–2C). We further explored the top five most common etiologies of cardiac readmissions. Among the ones identified, the incidence of non-ST elevation myocardial infarction (NSTEMI) was the highest in DKA patients (22.9%) compared to 13.6% in HHS patients (p = 0.021) and 14.3% in the non-DKA/HHS group (p = 0.042) (Fig 2D–2F). Interestingly, the incidence of STEMI in the HHS group was almost 2-fold higher than in the DKA group (37.3% vs. 20.8%, HHS vs. DKA, respectively, p<0.001) and similar when comparing between DKA and non-DKA/HHS group (20.8% vs. 23.9%, respectively, p = 0.096).

## IV. Discussion

We have shown that DKA and HHS are associated with higher mortality and morbidity in diabetes patients admitted for STEMI. Nevertheless, 30-day mortality was not significantly higher in both conditions. To our knowledge, our study is the first to compare the cardiovascular impact of two life-threatening hyperglycemic conditions in STEMI patients with diabetes.

Both DKA and HHS are acute metabolic disturbances characterized by hyperglycemia, indicating a decompensation in diabetes. DKA is defined by Hyperglycemia, elevated blood ketone levels, and high anion gap metabolic acidosis [12]. It most commonly occurs in patients with type 1 diabetes due to reduced insulin secretion. However, it can also develop in type 2 diabetics in the presence of infection, stress, and acute emergencies [13]. The underlying pathophysiology of DKA revolves around an overall state of hypoinsulinemia and heightened catabolism resulting from elevated glucagon, cortisol, catecholamines, and growth hormone levels. These hormonal changes promote glucose mobilization via gluconeogenesis and glycogenolysis (as well as proteolysis, which provides amino acids as substrates for gluconeogenesis), resulting in marked hyperglycemia. Additionally, lipolysis results in free fatty acid (FFA) release, which contributes to ketogenesis, resulting in ketonemia and high-anion gap metabolic acidosis.

In contrast, the pathophysiology of HHS differs. Patients with HHS, who typically have type 2 diabetes, often exhibit elevated insulin levels due to underlying insulin resistance. These higher insulin levels suppress ketogenesis. However, the combined effect of insulin resistance and elevated counter-regulatory hormones, such as glucagon, cortisol, and catecholamines, promotes glucose mobilization and downstream hyperglycemia. Both DKA and HHS patients experience dehydration caused by osmotic diuresis due to hyperglycemia. In addition, patients often demonstrate electrolyte abnormalities such as hypokalemia due to depleted intracellular potassium stores from hypoinsulinemia and potassium loss through urine from osmotic diuresis [13].

Acute hyperglycemic crises, such as DKA and HHS, may also increase cardiovascular risk due to the harmful and proinflammatory effects of hyperglycemia [14]. Previous studies have demonstrated that these hyperglycemic crises were associated with an elevation in proinflammatory markers such as IL-1B and -8 and nontraditional markers of cardiovascular risk. These markers include products of reactive oxygen species (ROS), C-reactive protein (CRP), homocysteine, and Plasma Activator Inhibitor-1 (PAI-1) [14]. PAI-1, in particular, may contribute to increased cardiovascular risk by impairing fibrinolysis and promoting hypercoagulability [15]. It has been recently shown that an oxidative mechanism mediates the increase in inflammatory cytokines [16]. Acute hyperglycemia also affects all major components of innate immunity, and specific distinctive proinflammatory alterations of the immune response can be observed under hyperglycemic conditions [17].

Our results are concordant with previous studies that reported a deleterious effect of hyperglycemia on myocardial infarction (MI). In a recent analysis of a STEMI cohort, Wei et al. reported higher odds of cardiovascular events with stress hyperglycemia in patients undergoing PCI, independently of the presence of diabetes [18], which was also present after two years of follow-up in a similar cohort [19]. Hyperglycemia has been found to correlate with larger infarct size and impaired left ventricular function [20]. In a cohort of 2704 patients with MI, Paolisso et al. demonstrated a significant relationship between blood glucose levels on admission and all-cause mortality [21]. Hyperglycemia has also been found to affect hemodynamic parameters, as elevated blood glucose was shown to increase mean heart rate and systolic and diastolic blood pressure readings [22].

On the other end, hypoglycemia is also associated with worse cardiovascular outcomes in MI patients. A retrospective database review showed that hypoglycemia was independently associated with an increased risk of mortality in critically ill patients in the ICU [23]. Another retrospective cohort study demonstrated that among diabetes patients admitted to general inpatient services, hypoglycemia was associated with an increased length of stay and higher mortality, both during the initial hospitalization and after discharge [24]. We have previously reported that hypoglycemia is associated with higher odds of in-hospital mortality and arrhythmia in STEMI patients [10]. The underlying pathophysiology that may account for such findings has been elucidated in other studies in which ischemic ECG changes were observed in patients with insulin-induced hypoglycemia [25]. Findings from different studies involving patients with hypoglycemia based on continuous glucose measurements reported symptoms of angina and demonstrated similar ischemic ECG findings [26]. Patients with diabetes exhibit an impaired counterregulatory response to hypoglycemic events, such as a suboptimal release of glucagon, cortisol, and epinephrine, which stimulate glycogenolysis and gluconeogenesis and inhibit insulin secretion [27]. These responses become dysfunctional in diabetic patients due to overexposure to hypoglycemic events. Importantly, hypoglycemia is responsible for increased cardiovascular risk due to its role in hemodynamic disturbances, ischemia of the myocardium, aberrancies in cardiac rhythm and electrophysiology, as well as promoting a prothrombotic and proinflammatory milieu [27], all of which can account for the increased odds of mortality with hypoglycemia.

It is unclear whether treating HHS and DKA decreases the cardiovascular risk. Several clinical trials have investigated the role of glucose control in patients with myocardial infarction. The first DIGAMI trial (DIGAMI-1) was conducted in the 1990s to investigate the effects of intensive insulin therapy in diabetes patients following MI [28]. The trial demonstrated that intensive insulin treatment significantly reduced mortality rates and recurrent myocardial infarctions in the immediate post-MI phase. The DIGAMI-II trial evaluated the long-term effects of different insulin regimens and other glucose-lowering medications on cardiovascular outcomes and overall survival [29]. However, the results contrast with the findings in the first DIGAMI trial. The CREATE-ECLA trial assessed the efficacy of glucose-insulin-potassium (GIK) infusion in reducing mortality and morbidity in acute myocardial infarction [30]. The study concluded that the routine use of GIK infusion does not improve outcomes. The HI-5 trial, which was published a year later, showed a trend toward reduced mortality with intensive glucose control, though not statistically significant [31]. However, an increased risk of hypoglycemia was observed with intensive therapy. Finally, the Normoglycemia in Intensive Care Evaluation—Survival Using Glucose Algorithm Regulation (NICE-SUGAR) trial, which assessed outcomes of intensive glucose control in critically ill patients, including those with myocardial infarction, showed that intensive glucose control increased mortality due to hypoglycemia [32].

We recognize the limitations inherent in our study. Our dataset is derived from an administrative database not initially designed for assessing cardiovascular events or establishing causal relationships. Further, the relatively small sample size for the readmission cohort of patients with DKA and HHS limited us from assessing time-to-first cardiovascular events. Furthermore, the lack of differences between the HHS and DKA groups might be attributable to an underpowered comparison, given the low prevalence of both conditions. This could also be due to an underreporting of both conditions. We could not rule out the misclassification of diabetes or the inclusion of patients with type 1 diabetes, which would explain the younger age of patients with DKA.

Additionally, several key parameters related to hyperglycemia, such as initial glycemia, baseline medications, HBA_1c_, duration of diabetes, and biochemical tests used to diagnose HHS and DKA, such as glycemia, pH, lactic acid, bicarbonate, and electrolytes, are absent from our analysis. These factors are known to be significant predictors of diabetes-related mortality, thus potentially impacting the comprehensiveness of our findings. Additionally, it is essential to acknowledge that all diagnoses and outcomes rely on the ICD-10 coding systems. Therefore, the possibility of erroneous coding or misclassification cannot be discounted.

## V. Conclusion

In the analysis on a large cohort, the presence of diabetic ketoacidosis or hyperglycemic hyperosmolar states is associated with higher odds of in-hospital mortality, cardiogenic shock, acute renal failure, and 30-day readmission but not mortality in patients with type 2 diabetes who are hospitalized for ST-elevation myocardial infarction. Future directions should aim at developing and validating risk stratification tools to identify patients with type 2 diabetes who are at higher risk of complications such as diabetic ketoacidosis (DKA) or hyperglycemic hyperosmolar state (HHS) during hospitalization for ST-elevation myocardial infarction (STEMI). Tailored glycemic management protocols for patients with diabetes, especially those with a history of DKA or HHS, could minimize the occurrence of acute hyperglycemic crises and lower the incidence of cardiovascular complications.
